# Supplementary material for: Learning to kill: Why a small handful of counties generates the bulk of US death sentences
Source: PLoS One. 2020 Oct 27;15(10):e0240401. doi: 10.1371/journal.pone.0240401 (PMC7591063; doi:10.1371/journal.pone.0240401)
Supplement: S1 File — (DOCX) [file pone.0240401.s001.docx]

# Learning to Kill:

# Why a Small Handful of Counties Generates the Bulk of US Death Sentences

# *PLoS-ONE*

# Frank R. Baumgartner, Janet M. Box-Steffensmeier, Benjamin W. Campbell, Christian Caron, and Hailey Sherman

# October 1, 2020

# Supplemental File 1: Alternative ZINB models

In order to test the robustness of the effect of previous cumulative death sentences on predicting death sentences, we conduct a series of models with alternate specifications of variables and county filters. Across the tests, the effect of previous cumulative death sentences remains stable and statistically significant.

Given the variance in county population size, and thus total number of homicides and death sentences, we first run the full ZINB model on subsets of the data, based on both county population size and homicide counts. Table S1 lists the results specifically for the effect of previous cumulative death sentences by each county subset, and the full model results are presented in Tables S3 and S4. The stricter filters on counties do slightly decrease the value of the coefficient, but it remains generally consistent.

We also confirm the robustness of our analysis with additional variables and alternate measurements of variables. Table S2 contains the condensed results, with the full models in Tables S5-S8. To confirm that there is not a longer lagged effect of homicides, we include the homicide variable lagged at two, three, four, and five years in place of the homicides*_t-1_* variable. Based on the research of Jacobs, Carmichael, and Kent (2005) and Jacobs and Carmichael (2002), we run models that include an interaction between racial threat and lynchings, the violent and property crime rates, a measure of evangelicalism, the unemployment rate, percent in poverty, and an indicator about the partisanship of the legislature. Additionally, we substitute in other measures of variables, such as different estimates of the lynching variable, to ensure that the effect is not due to the specific variables used in the full model. These results also provide evidence of the robustness of the effect of previous cumulative death sentences as the coefficients remain stable and statistically significant.

Table S9 presents the results from our original model alongside those from models specified with two alternative inertia variables: prior cumulative executions and one-year lagged executions. Overall, the results suggest that states’ execution histories are not driving our results. The effect of previous cumulative executions fails to reach statistical significance when substituting this variable for previous cumulative death sentences. Additionally, although executions appear to have a short-term, positive influence on the number of death sentence issued in a county in one of the models, this effect dissipates after accounting for prior cumulative death sentences, which continues to exert a large and statistically significant effect.

Table S1. Predicting Death Sentences with Previous Cumulative Death Sentence

|  | IRR for  Cumulative Death Sentences_t-1_ | Robust Standard  Error for  Cumulative Death Sentences_t-1_ | |
| --- | --- | --- | --- |
| Counties with Population Size > 10,000 | 1.08*** | (0.03) |  |
| Counties with Population Size > 25,000 | 1.08** | (0.03) |  |
| Counties with Population Size > 50,000 | 1.08** | (0.03) |  |
| Counties with Population Size > 100,000 | 1.07** | (0.02) |  |
| Counties with Homicide Count > 4 | 1.07** | (0.02) |  |
| Counties with Homicide Count > 9 | 1.07*** | (0.02) |  |
| Counties with Homicide Count > 24 | 1.06*** | (0.02) |  |
| Counties with Homicide Count > 49 | 1.05*** | (0.02) |  |

***p < 0.001, **p < 0.01, *p < 0.05

Table S2. Predicting Death Sentences with Alternate Models

| County | IRR for  Cumulative Death Sentences_t-1_ | Robust Standard  Error for  Cumulative Death Sentences_t-1_ |  |  |
| --- | --- | --- | --- | --- |
| Model with Homicides*_t-2_* | 1.09*** | (0.03) |  |  |
| Model with Homicides*_t-3_* | 1.09*** | (0.03) |  |  |
| Model with Homicides*_t-4_* | 1.09*** | (0.03) |  |  |
| Model with Homicides*_t-5_* | 1.09*** | (0.03) |  |  |
| Model with Threat*Lynching Interaction | 1.09*** | (0.03) |  |  |
| Model with Violent Crime Rate | 1.09*** | (0.03) |  |  |
| Model with Violent Crime Rate and Property Crime Rate | 1.09*** | (0.03) |  |  |
| Model with Percent Evangelical | 1.09*** | (0.03) |  |  |
| Model with Republican Legislature | 1.09*** | (0.03) |  |  |
| Model with Homicide Rate | 1.09*** | (0.03) |  |  |
| Model with White Population | 1.08*** | (0.03) |  |  |
| Model with Black Population | 1.08*** | (0.03) |  |  |
| Model with Poverty | 1.08*** | (0.02) |  |  |
| Model with Unemployment | 1.08*** | (0.02) |  |  |
| Model with Culture | 1.09*** | (0.03) |  |  |
| Model with Black Lynching (Alternate Measure) | 1.09*** | (0.03) |  |  |
| Model with Black Lynching (*This Cruel War*) | 1.09*** | (0.03) |  |  |
| Model with Total Lynching (*This Cruel War*) | 1.09*** | (0.03) |  |  |
| Model with Percent Slave | 1.09*** | (0.03) |  |  |

***p < 0.001, **p < 0.01, *p < 0.05

Table S3. Model Results – Varying Population Sizes

|  | Counties with Population >10,000 | Counties with Population >25,000 | Counties with Population >50,000 | Counties with Population >100,000 |
| --- | --- | --- | --- | --- |
| *Death Sentence Absence* |  |  |  |  |
| Cumulative Death Sentences*_t-1_*/10 | 0.03*** | 0.03*** | 0.04*** | 0.05*** |
|  | (0.02) | (0.02) | (0.02) | (0.04) |
| Homicides*_t-1_* /100 | 1.06 | 1.04 | 1.04 | 1.01 |
|  | (0.07) | (0.07) | (0.07) | (0.07) |
| Racial Threat/100 | 1.01 | 0.89 | 1.07 | 0.75 |
|  | (0.84) | (0.89) | (1.24) | (1.10) |
| Lynchings/10 | 1.12 | 1.26 | 1.28 | 1.16 |
|  | (0.22) | (0.26) | (0.30) | (0.47) |
| Ln Population | 0.76*** | 0.84 | 0.90 | 1.13 |
|  | (0.06) | (0.08) | (0.10) | (0.17) |
| Republican Governor | 0.91 | 0.91 | 0.88 | 0.86 |
|  | (0.11) | (0.12) | (0.13) | (0.15) |
| Partisan Supreme Court Elections | 0.74* | 0.68* | 0.61* | 0.52* |
|  | (0.11) | (0.12) | (0.13) | (0.15) |
| Citizen Ideology/100 | 3.70 | 5.76 | 3.93 | 3.64 |
|  | (3.86) | (7.03) | (5.46) | (5.53) |
| South | 0.63* | 0.72 | 0.68 | 0.76 |
|  | (0.11) | (0.15) | (0.16) | (0.22) |
| Constant | 47.49*** | 11.46* | 4.22 | 0.20 |
|  | (0.99) | (1.18) | (1.46) | (2.04) |
| Year FE | Yes | Yes | Yes | Yes |
| *One or more Death Sentences* |  |  |  |  |
| Cumulative Death Sentences*_t-1_*/10 | 1.08*** | 1.08** | 1.08** | 1.07** |
|  | (0.03) | (0.03) | (0.03) | (0.02) |
| Homicides*_t-1_* /100 | 1.00 | 1.00 | 1.00 | 1.00 |
|  | (0.02) | (0.02) | (0.02) | (0.02) |
| Racial Threat/100 | 3.04* | 3.10* | 3.38* | 3.21* |
|  | (1.48) | (1.62) | (1.84) | (1.84) |
| Lynchings/10 | 1.27*** | 1.29*** | 1.29*** | 1.30*** |
|  | (0.09) | (0.09) | (0.09) | (0.10) |
| Ln Population | 1.93*** | 1.97*** | 1.95*** | 2.01*** |
|  | (0.09) | (0.10) | (0.11) | (0.12) |
| Republican Governor | 1.00 | 1.00 | 0.99 | 0.97 |
|  | (0.06) | (0.06) | (0.06) | (0.07) |
| Partisan Supreme Court Elections | 1.00 | 0.99 | 0.98 | 0.96 |
|  | (0.09) | (0.09) | (0.10) | (0.11) |
| Citizen Ideology/100 | 0.32 | 0.32 | 0.30 | 0.25 |
|  | (0.21) | (0.22) | (0.22) | (0.18) |
| South | 1.00 | 1.01 | 1.00 | 0.97 |
|  | (0.11) | (0.12) | (0.12) | (0.12) |
| Constant | 0.00*** | 0.00*** | 0.00*** | 0.00*** |
|  | (0.00) | (0.00) | (0.00) | (0.00) |
| Year FE | Yes | Yes | Yes | Yes |
| Total obs. | 78,647 | 49,205 | 28,816 | 16,371 |
| Nonzero obs. | 4,968 | 4,561 | 3,967 | 3,261 |
| Log pseudolikelihood | -17096.93 | -14971.05 | -12302.29 | -9547.33 |

***p < 0.001, **p < 0.01, *p < 0.05 Notes: Reported values are incidence rate ratios for the negative binomial model and odds ratios for the logit model. Robust standard errors clustered by county in parentheses. Each variable is rescaled by the factor indicated in order to generate coefficients that can be more easily interpreted. Models specified with year fixed effects.

Table S4. Model Results – Varying Homicide Counts

|  | Counties with Homicide Count > 4 | Counties with Homicide Count > 9 | Counties with Homicide Count > 24 | Counties with Homicide Count > 49 |
| --- | --- | --- | --- | --- |
| *Death Sentence Absence* |  |  |  |  |
| Cumulative Death Sentences*_t-1_*/10 | 0.05*** | 0.05*** | 0.17*** | 0.22*** |
|  | (0.04) | (0.04) | (0.07) | (0.07) |
| Homicides*_t-1_* /100 | 1.03 | 1.04 | 1.05 | 1.10 |
|  | (0.07) | (0.07) | (0.08) | (0.08) |
| Racial Threat/100 | 4.08 | 1.07 | 0.04 | 0.65 |
|  | (5.35) | (1.67) | (0.09) | (1.29) |
| Lynchings/10 | 1.33 | 1.37 | 0.65 | 0.13 |
|  | (0.31) | (0.41) | (0.33) | (0.20) |
| Ln Population | 1.00 | 1.07 | 1.12 | 0.78 |
|  | (0.15) | (0.19) | (0.29) | (0.29) |
| Republican Governor | 0.87 | 0.76 | 0.50* | 0.39* |
|  | (0.17) | (0.16) | (0.14) | (0.17) |
| Partisan Supreme Court Elections | 0.67 | 0.56 | 0.62 | 1.11 |
|  | (0.17) | (0.17) | (0.33) | (0.63) |
| Citizen Ideology/100 | 5.22 | 5.61 | 2.55 | 1.26 |
|  | (8.43) | (9.21) | (4.31) | (3.33) |
| South | 0.74 | 0.81 | 0.79 | 0.83 |
|  | (0.20) | (0.25) | (0.40) | (0.65) |
| Constant | 0.22 | 0.20 | 1.46 | 13.47 |
|  | (2.14) | (2.42) | (3.95) | (7.72) |
| Year FE | Yes | Yes | Yes | Yes |
| *One or more Death Sentences* |  |  |  |  |
| Cumulative Death Sentences*_t-1_*/10 | 1.07** | 1.07*** | 1.06*** | 1.05*** |
|  | (0.02) | (0.02) | (0.02) | (0.02) |
| Homicides*_t-1_* /100 | 1.00 | 1.00 | 0.99 | 1.00 |
|  | (0.02) | (0.02) | (0.02) | (0.02) |
| Racial Threat/100 | 3.295* | 2.50 | 2.01 | 1.96 |
|  | (1.84) | (1.46) | (1.34) | (1.39) |
| Lynchings/10 | 1.30*** | 1.30*** | 1.28*** | 1.15 |
|  | (0.09) | (0.09) | (0.09) | (0.12) |
| Ln Population | 2.00*** | 2.00*** | 2.00*** | 1.91*** |
|  | (0.12) | (0.13) | (0.17) | (0.26) |
| Republican Governor | 0.97 | 0.94 | 0.88 | 0.89 |
|  | (0.07) | (0.07) | (0.07) | (0.09) |
| Partisan Supreme Court Elections | 0.98 | 0.99 | 1.02 | 1.08 |
|  | (0.11) | (0.11) | (0.16) | (0.19) |
| Citizen Ideology/100 | 0.29 | 0.23* | 0.22* | 0.13** |
|  | (0.21) | (0.16) | (0.16) | (0.10) |
| South | 0.98 | 0.91 | 0.86 | 0.78 |
|  | (0.12) | (0.12) | (0.12) | (0.11) |
| Constant | 0.00*** | 0.00*** | 0.00*** | 0.00*** |
|  | (0.00) | (0.00) | (0.00) | (0.00) |
| Year FE | Yes | Yes | Yes | Yes |
| Total obs. | 19,721 | 9,603 | 4,537 | 2,532 |
| Nonzero obs. | 3,552 | 2,683 | 1,803 | 1,217 |
| Log pseudolikelihood | -10610.74 | -7439.06 | -4721.42 | -3156.43 |

***p < 0.001, **p < 0.01, *p < 0.05 Notes: Reported values are incidence rate ratios for the negative binomial model and odds ratios for the logit model. Robust standard errors clustered by county in parentheses. Each variable is rescaled by the factor indicated in order to generate coefficients that can be more easily interpreted. Models specified with year fixed effects.

Table S5. Model Results – Homicide Lags

|  | Homicides Lagged 2 Years | Homicides Lagged 3 Years | Homicides Lagged 4 Years | Homicides Lagged 5 Years |
| --- | --- | --- | --- | --- |
| *Death Sentence Absence* |  |  |  |  |
| Cumulative Death Sentences*_t-1_*/10 | 0.03*** | 0.03*** | 0.03*** | 0.03*** |
|  | (0.02) | (0.02) | (0.02) | (0.02) |
| Homicides*_t-2_* /100 | 1.09 |  |  |  |
|  | (0.07) |  |  |  |
| Homicides*_t-3_* /100 |  | 1.10 |  |  |
|  |  | (0.08) |  |  |
| Homicides*_t-4_* /100 |  |  | 1.11 |  |
|  |  |  | (0.07) |  |
| Homicides*_t-5_* /100 |  |  |  | 1.13 |
|  |  |  |  | (0.08) |
| Racial Threat/100 | 0.82 | 0.79 | 0.80 | 0.78 |
|  | (0.65) | (0.63) | (0.64) | (0.64) |
| Lynchings/10 | 1.12 | 1.12 | 1.12 | 1.08 |
|  | (0.22) | (0.22) | (0.22) | (0.22) |
| Ln Population | 0.72*** | 0.73*** | 0.73*** | 0.75*** |
|  | (0.05) | (0.05) | (0.05) | (0.05) |
| Republican Governor | 0.94 | 0.94 | 0.95 | 0.90 |
|  | (0.11) | (0.11) | (0.11) | (0.11) |
| Partisan Supreme Court Elections | 0.74* | 0.75* | 0.75* | 0.76 |
|  | (0.11) | (0.11) | (0.11) | (0.12) |
| Citizen Ideology/100 | 3.96 | 3.98 | 3.97 | 3.55 |
|  | (4.02) | (4.05) | (4.04) | (3.66) |
| South | 0.63** | 0.63** | 0.63** | 0.61** |
|  | (0.11) | (0.11) | (0.11) | (0.11) |
| Constant | 82.89*** | 81.08*** | 66.48*** | 40.12*** |
|  | (0.92) | (0.92) | (0.92) | (0.92) |
| Year FE | Yes | Yes | Yes | Yes |
| *One or more Death Sentences* |  |  |  |  |
| Cumulative Death Sentences*_t-1_*/10 | 1.09*** | 1.09** | 1.09** | 1.09** |
|  | (0.03) | (0.03) | (0.03) | (0.03) |
| Homicides*_t-2_* /100 | 1.00 |  |  |  |
|  | (0.02) |  |  |  |
| Homicides*_t-3_* /100 |  | 1.00 |  |  |
|  |  | (0.02) |  |  |
| Homicides*_t-4_* /100 |  |  | 1.00 |  |
|  |  |  | (0.02) |  |
| Homicides*_t-5_* /100 |  |  |  | 0.99 |
|  |  |  |  | (0.02) |
| Racial Threat/100 | 3.00* | 2.97* | 2.96* | 2.87* |
|  | (1.42) | (1.40) | (1.40) | (1.37) |
| Lynchings/10 | 1.27*** | 1.27*** | 1.27*** | 1.27** |
|  | (0.09) | (0.09) | (0.09) | (0.09) |
| Ln Population | 1.92*** | 1.92*** | 1.93*** | 1.95*** |
|  | (0.08) | (0.08) | (0.08) | (0.09) |
| Republican Governor | 1.01 | 1.01 | 1.01 | 0.99 |
|  | (0.06) | (0.06) | (0.06) | (0.06) |
| Partisan Supreme Court Elections | 1.00 | 1.00 | 1.00 | 1.01 |
|  | (0.09) | (0.09) | (0.09) | (0.09) |
| Citizen Ideology/100 | 0.32 | 0.33 | 0.33 | 0.31 |
|  | (0.21) | (0.21) | (0.21) | (0.20) |
| South | 1.00 | 1.00 | 1.00 | 0.99 |
|  | (0.11) | (0.11) | (0.11) | (0.11) |
| Constant | 0.00*** | 0.00*** | 0.00*** | 0.00*** |
|  | (0.00) | (0.00) | (0.00) | (0.00) |
| Year FE | Yes | Yes | Yes | Yes |
| Total obs. | 102,057 | 102,049 | 102,042 | 99,818 |
| Nonzero obs. | 5,057 | 5,057 | 5,056 | 4,929 |
| Log pseudolikelihood | -17651.75 | -17650.79 | -17645.63 | -17156.09 |

***p < 0.001, **p < 0.01, *p < 0.05 Notes: Reported Values are Incidence Rate Ratios. Robust standard errors clustered by county in parentheses. Each variable is rescaled by the factor indicated in order to generate coefficients that can be more easily interpreted. Models specified with year fixed effects.

Table S6. Model Results – Additional Variables

|  | Full Model with Threat*  Lynching Interaction | Full Model with Violent Crime Rate | Full Model with Violent Crime Rate and Property Crime Rate | Full Model with Percent Evangelical | Full Model with Republican Legislature |
| --- | --- | --- | --- | --- | --- |
| *Death Sentence Absence* |  |  |  |  |  |
| Cumulative Death Sentences*_t-1_*/10 | 0.03*** | 0.03*** | 0.03*** | 0.03*** | 0.03*** |
|  | (0.02) | (0.02) | (0.02) | (0.01) | (0.01) |
| Homicides*_t-1_* /100 | 1.08 | 1.08 | 1.08 | 1.08 | 1.07 |
|  | (0.07) | (0.08) | (0.07) | (0.07) | (0.08) |
| Racial Threat/100 | 0.65 | 1.19 | 1.31 | 0.44 | 0.93 |
|  | (0.67) | (0.95) | (1.05) | (0.39) | (0.75) |
| Lynchings/10 | 0.27 | 1.16 | 1.14 | 1.17 | 1.17 |
|  | (0.45) | (0.23) | (0.24) | (0.21) | (0.23) |
| Ln Population | 0.72*** | 0.71*** | 0.70*** | 0.74*** | 0.73*** |
|  | (0.05) | (0.05) | (0.05) | (0.05) | (0.05) |
| Republican Governor | 0.93 | 0.94 | 0.94 | 0.94 | 0.90 |
|  | (0.11) | (0.11) | (0.11) | (0.11) | (0.10) |
| Partisan Supreme Court Elections | 0.74* | 0.80 | 0.77 | 0.80 | 0.77 |
|  | (0.11) | (0.13) | (0.13) | (0.13) | (0.12) |
| Citizen Ideology/100 | 4.03 | 6.07 | 6.10 | 5.53 | 3.05 |
|  | (4.18) | (6.22) | (5.93) | (5.87) | (2.93) |
| South | 0.64** | 0.63* | 0.62* | 0.66* | 0.66* |
|  | (0.11) | (0.12) | (0.12) | (0.11) | (0.11) |
| Racial Threat * Lynching | 5.11 |  |  |  |  |
|  | (9.85) |  |  |  |  |
| Violent Crime Rate |  | 1.00 | 1.00 |  |  |
|  |  | (0.00) | (0.00) |  |  |
| Property Crime Rate |  |  | 1.00 |  |  |
|  |  |  | (0.00) |  |  |
| Percent Evangelical |  |  |  | 1.00 |  |
|  |  |  |  | (0.01) |  |
| Republican Legislature |  |  |  |  | 2.16 |
|  |  |  |  |  | (1.41) |
| Constant | 120.59*** | 97.49*** | 129.36*** | 93.07*** | 69.37*** |
|  | (1.03) | (0.92) | (0.95) | (1.00) | (0.96) |
| Year FE | Yes | Yes | Yes | Yes | Yes |
| *One or more Death Sentences* |  |  |  |  |  |
| Cumulative Death Sentences*_t-1_*/10 | 1.09*** | 1.09*** | 1.09*** | 1.09*** | 1.08*** |
|  | (0.03) | (0.03) | (0.03) | (0.03) | (0.03) |
| Homicides*_t-1_* /100 | 1.00 | 1.00 | 1.00 | 0.99 | 1.00 |
|  | (0.02) | (0.02) | (0.02) | (0.02) | (0.02) |
| Racial Threat/100 | 3.41* | 3.01* | 2.98* | 2.18 | 2.95* |
|  | (2.12) | (1.40) | (1.39) | (1.09) | (1.39) |
| Lynchings/10 | 1.56 | 1.26** | 1.27** | 1.21** | 1.28*** |
|  | (1.08) | (0.09) | (0.10) | (0.08) | (0.10) |
| Ln Population | 1.91*** | 1.90*** | 1.89*** | 2.05*** | 1.91*** |
|  | (0.09) | (0.09) | (0.09) | (0.09) | (0.08) |
| Republican Governor | 1.01 | 1.00 | 1.00 | 1.02 | 1.00 |
|  | (0.06) | (0.06) | (0.06) | (0.06) | (0.06) |
| Partisan Supreme Court Elections | 1.00 | 1.06 | 1.06 | 0.99 | 1.00 |
|  | (0.09) | (0.10) | (0.10) | (0.09) | (0.09) |
| Citizen Ideology/100 | 0.33 | 0.28* | 0.32 | 0.40 | 0.26* |
|  | (0.22) | (0.18) | (0.19) | (0.25) | (0.16) |
| South | 1.00 | 0.93 | 0.93 | 0.95 | 0.98 |
|  | (0.11) | (0.11) | (0.11) | (0.10) | (0.10) |
| Racial Threat * Lynching | 0.80 |  |  |  |  |
|  | (0.64) |  |  |  |  |
| Violent Crime Rate |  | 1.00 | 1.00 |  |  |
|  |  | (0.00) | (0.00) |  |  |
| Property Crime Rate |  |  | 1.00 |  |  |
|  |  |  | (0.00) |  |  |
| Percent Evangelical |  |  |  | 1.01* |  |
|  |  |  |  | (0.00) |  |
| Republican Legislature |  |  |  |  | 1.30 |
|  |  |  |  |  | (0.54) |
| Constant | 0.00*** | 0.00*** | 0.00*** | 0.00*** | 0.00*** |
|  | (0.00) | (0.00) | (0.00) | (0.00) | (0.00) |
| Year FE | Yes | Yes | Yes | Yes | Yes |
| Total obs. | 102,065 | 102,065 | 102,065 | 84,146 | 98,252 |
| Nonzero obs. | 5,057 | 5,057 | 5,057 | 4,594 | 5,028 |
| Log pseudolikelihood | -17650.15 | -17630.71 | -17624.53 | -15888.04 | -17517.06 |

***p < 0.001, **p < 0.01, *p < 0.05 Notes: Reported Values are Incidence Rate Ratios. Robust standard errors clustered by county in parentheses. Each variable is rescaled by the factor indicated in order to generate coefficients that can be more easily interpreted. Models specified with year fixed effects.

Table S7. Model Results – Alternative and Additional Variables

|  | Model with Homicide Rate | Model with White Population | Model with Black Population | Model with Poverty | Model with Unemployment | Model with Culture |
| --- | --- | --- | --- | --- | --- | --- |
| *Death Sentence Absence* |  |  |  |  |  |  |
| Cumulative Death Sentences*_t-1_*/10 | 0.03*** | 0.03*** | 0.03*** | 0.03*** | 0.03*** | 0.03*** |
|  | (0.02) | (0.02) | (0.02) | (0.02) | (0.02) | (0.02) |
| Homicides*_t-1_* /100 |  | 1.08 | 1.08 | 1.05 | 1.08 | 1.08 |
|  |  | (0.08) | (0.07) | (0.07) | (0.07) | (0.07) |
| Racial Threat/100 | 2.34 |  |  | 0.71 | 0.85 | 0.83 |
|  | (1.91) |  |  | (0.55) | (0.68) | (0.67) |
| Lynchings/10 | 1.21 | 1.05 | 1.21 | 1.03 | 1.12 | 1.18 |
|  | (0.25) | (0.21) | (0.25) | (0.19) | (0.21) | (0.22) |
| Ln Population | 0.70*** | 0.74*** | 0.75*** | 0.77*** | 0.73*** | 0.71*** |
|  | (0.05) | (0.05) | (0.05) | (0.06) | (0.05) | (0.05) |
| Republican Governor | 0.96 | 0.96 | 0.96 | 0.95 | 0.93 | 0.96 |
|  | (0.11) | (0.11) | (0.11) | (0.11) | (0.11) | (0.11) |
| Partisan Supreme Court Elections | 0.74* | 0.73* | 0.72* | 0.72* | 0.75* | 0.72* |
|  | (0.11) | (0.10) | (0.10) | (0.10) | (0.11) | (0.10) |
| Citizen Ideology/100 | 2.56 | 3.50 | 4.08 | 4.52 | 3.27 | 3.97 |
|  | (2.70) | (3.57) | (4.17) | (4.76) | (3.30) | (4.12) |
| South | 0.65* | 0.62** | 0.66* | 0.62** | 0.65* |  |
|  | (0.11) | (0.11) | (0.13) | (0.11) | (0.11) |  |
| Homicide Rate | 0.01* |  |  |  |  |  |
|  | (0.02) |  |  |  |  |  |
| White Population |  | 1.00 |  |  |  |  |
|  |  | (0.01) |  |  |  |  |
| Percent Black |  |  | 0.99 |  |  |  |
|  |  |  | (0.01) |  |  |  |
| Percent Black * Percent Black |  |  | 1.00 |  |  |  |
|  |  |  | (0.00) |  |  |  |
| Percent in Poverty |  |  |  | 1.05** |  |  |
|  |  |  |  | (0.02) |  |  |
| Unemployment |  |  |  |  | 1.03 |  |
|  |  |  |  |  | (0.04) |  |
| Culture |  |  |  |  |  | 0.88** |
|  |  |  |  |  |  | (0.04) |
| Constant | 112.37*** | 49.42*** | 57.82*** | 25.58** | 73.55*** | 228.29*** |
|  | (0.98) | (0.94) | (0.89) | (1.11) | (1.06) | (1.04) |
| Year FE | Yes | Yes | Yes | Yes | Yes | Yes |
| *One or more Death Sentences* |  |  |  |  |  |  |
| Cumulative Death Sentences*_t-1_*/10 | 1.09*** | 1.08** | 1.08** | 1.08*** | 1.08*** | 1.09*** |
|  | (0.03) | (0.03) | (0.03) | (0.02) | (0.02) | (0.03) |
| Homicides*_t-1_* /100 |  | 1.00 | 1.00 | 0.99 | 1.00 | 1.00 |
|  |  | (0.02) | (0.02) | (0.02) | (0.02) | (0.02) |
| Racial Threat/100 | 3.48** |  |  | 2.75* | 3.03* | 2.86* |
|  | (1.63) |  |  | (1.31) | (1.43) | (1.34) |
| Lynchings/10 | 1.28** | 1.24** | 1.28** | 1.22** | 1.23** | 1.24** |
|  | (0.09) | (0.09) | (0.10) | (0.09) | (0.09) | (0.09) |
| Ln Population | 1.92*** | 1.98*** | 1.97*** | 1.98*** | 1.96*** | 1.96*** |
|  | (0.09) | (0.08) | (0.09) | (0.09) | (0.09) | (0.08) |
| Republican Governor | 1.03 | 1.02 | 1.03 | 1.01 | 1.00 | 1.04 |
|  | (0.06) | (0.06) | (0.06) | (0.06) | (0.06) | (0.06) |
| Partisan Supreme Court Elections | 1.00 | 1.00 | 1.00 | 0.98 | 1.00 | 0.98 |
|  | (0.09) | (0.08) | (0.08) | (0.08) | (0.08) | (0.08) |
| Citizen Ideology/100 | 0.28* | 0.28 | 0.28* | 0.34 | 0.26* | 0.46 |
|  | (0.18) | (0.18) | (0.18) | (0.22) | (0.17) | (0.29) |
| South | 0.99 | 1.02 | 0.95 | 1.00 | 1.03 |  |
|  | (0.11) | (0.12) | (0.11) | (0.11) | (0.12) |  |
| Homicide Rate | 1.12 |  |  |  |  |  |
|  | (0.67) |  |  |  |  |  |
| White Population |  | 1.00 |  |  |  |  |
|  |  | (0.00) |  |  |  |  |
| Percent Black |  |  | 1.02* |  |  |  |
|  |  |  | (0.01) |  |  |  |
| Percent Black * Percent Black |  |  | 1.00* |  |  |  |
|  |  |  | (0.00) |  |  |  |
| Percent in Poverty |  |  |  | 1.03* |  |  |
|  |  |  |  | (0.01) |  |  |
| Unemployment |  |  |  |  | 1.05* |  |
|  |  |  |  |  | (0.02) |  |
| Culture |  |  |  |  |  | 1.04 |
|  |  |  |  |  |  | (0.03) |
| Constant | 0.00*** | 0.00*** | 0.00*** | 0.00*** | 0.00*** | 0.00*** |
|  | (0.00) | (0.00) | (0.00) | (0.00) | (0.00) | (0.00) |
| Year FE | Yes | Yes | Yes | Yes | Yes | Yes |
| Total obs. | 102,064 | 102,065 | 101,998 | 102,065 | 102,065 | 97,347 |
| Nonzero obs. | 5,057 | 5,057 | 5,034 | 5,057 | 5,057 | 4,995 |
| Log pseudo-likelihood | -17615.25 | -17671.93 | -96,964 | -17639 | -17639.39 | -17363.29 |

***p < 0.001, **p < 0.01, *p < 0.05 Notes: Reported Values are Incidence Rate Ratios for the negative binomial model and Odds Ratios for the logit model. Robust standard errors clustered by county in parentheses. Each variable is rescaled by the factor indicated in order to generate coefficients that can be more easily interpreted. Models specified with year fixed effects.

Table S8. Model Results – Alternative Lynching Variables

|  | Model with Black Lynching (Alternate Measure) | Model with Black Lynching (*This Cruel War*) | Model with Total Lynching (*This Cruel War*) | Model with Percent Slave |
| --- | --- | --- | --- | --- |
| *Death Sentence Absence* |  |  |  |  |
| Cumulative Death Sentences*_t-1_*/10 | 0.03*** | 0.03*** | 0.03*** | 0.03*** |
|  | (0.02) | (0.02) | (0.02) | (0.02) |
| Homicides*_t-1_* /100 | 1.08 | 1.08 | 1.08 | 1.08 |
|  | (0.07) | (0.07) | (0.07) | (0.07) |
| Racial Threat/100 | 0.81 | 0.84 | 0.95 | 0.67 |
|  | (0.64) | (0.67) | (0.76) | (0.53) |
| Ln Population | 0.73*** | 0.73*** | 0.72*** | 0.73*** |
|  | (0.05) | (0.05) | (0.05) | (0.05) |
| Republican Governor | 0.94 | 0.94 | 0.94 | 0.95 |
|  | (0.11) | (0.11) | (0.11) | (0.11) |
| Partisan Supreme Court Elections | 0.76 | 0.76 | 0.76 | 0.74* |
|  | (0.11) | (0.11) | (0.11) | (0.11) |
| Citizen Ideology/100 | 3.48 | 3.66 | 4.08 | 3.13 |
|  | (3.52) | (3.71) | (4.13) | (3.15) |
| South | 0.63** | 0.64* | 0.62** | 0.70 |
|  | (0.11) | (0.12) | (0.11) | (0.14) |
| Black Lynching (Alternate Measure)/10 | 1.25 |  |  |  |
|  | (0.22) |  |  |  |
| Black Lynchings (*This Cruel War*)/10 |  | 1.10 |  |  |
|  |  | (0.20) |  |  |
| Total Lynchings (*This Cruel War*)/10 |  |  | 1.13 |  |
|  |  |  | (0.17) |  |
| Percent Slavery |  |  |  | 0.95 |
|  |  |  |  | (0.45) |
| Constant | 101.45*** | 97.35*** | 93.30*** | 115.90*** |
|  | (0.93) | (0.92) | (0.93) | (0.93) |
| Year FE | Yes | Yes | Yes | Yes |
| *One or more Death Sentences* |  |  |  |  |
| Cumulative Death Sentences*_t-1_*/10 | 1.09*** | 1.09*** | 1.09*** | 1.09** |
|  | (0.03) | (0.03) | (0.03) | (0.03) |
| Homicides*_t-1_* /100 | 1.01 | 1.00 | 1.01 | 1.00 |
|  | (0.02) | (0.02) | (0.02) | (0.02) |
| Racial Threat/100 | 3.06* | 3.07* | 3.40* | 2.68* |
|  | (1.45) | (1.47) | (1.64) | (1.28) |
| Ln Population | 1.92*** | 1.92*** | 1.90*** | 1.92*** |
|  | (0.08) | (0.08) | (0.08) | (0.09) |
| Republican Governor | 1.01 | 1.01 | 1.01 | 1.01 |
|  | (0.06) | (0.06) | (0.06) | (0.06) |
| Partisan Supreme Court Elections | 1.02 | 1.01 | 1.01 | 1.01 |
|  | (0.09) | (0.09) | (0.09) | (0.09) |
| Citizen Ideology/100 | 0.29 | 0.31 | 0.33 | 0.27* |
|  | (0.19) | (0.20) | (0.22) | (0.17) |
| South | 1.00 | 1.00 | 0.99 | 1.06 |
|  | (0.11) | (0.11) | (0.11) | (0.13) |
| Black Lynching (Alternate Measure)/10 | 1.33*** |  |  |  |
|  | (0.09) |  |  |  |
| Black Lynchings (*This Cruel War*)/10 |  | 1.23** |  |  |
|  |  | (0.09) |  |  |
| Total Lynchings (*This Cruel War*)/10 |  |  | 1.22*** |  |
|  |  |  | (0.07) |  |
| Percent Slavery |  |  |  | 1.10 |
|  |  |  |  | (0.29) |
| Constant | 0.00*** | 0.00*** | 0.00*** | 0.00*** |
|  | (0.00) | (0.00) | (0.00) | (0.00) |
| Year FE | Yes | Yes | Yes | Yes |
| Total obs. | 102,065 | 102,065 | 102,065 | 102,065 |
| Nonzero obs. | 5,057 | 5,057 | 5,057 | 5,057 |
| Log pseudolikelihood | -17654.08 | -17655.8 | -17654.87 | -17672.75 |

***p < 0.001, **p < 0.01, *p < 0.05 Notes: Reported Values are Incidence Rate Ratios. Robust standard errors clustered by county in parentheses. Each variable is rescaled by the factor indicated in order to generate coefficients that can be more easily interpreted. Models specified with year fixed effects.

Table S9. Model Results – Alternative Inertia Variables

|  | Model with Cumulative Death Sentences  (Same as in main text) | Model with Cumulative Executions | Model with Lagged Executions | Model with Cumulative Death Sentences and Lagged Executions |
| --- | --- | --- | --- | --- |
| *Death Sentence Absence* |  |  |  |  |
| Cumulative Death Sentences*_t-1_*/10 | 0.03*** |  |  | 0.03*** |
|  | (0.02) |  |  | (0.02) |
| Homicides*_t-1_*/100 | 1.07 | 0.27 | 0.25* | 1.09 |
|  | (0.07) | (0.20) | (0.16) | (0.07) |
| Racial Threat/100 | 0.84 | 0.00** | 0.00* | 0.83 |
|  | (0.67) | (0.00) | (0.00) | (0.68) |
| Lynchings/10 | 1.12 | 0.45 | 0.36 | 1.14 |
|  | (0.22) | (1.27) | (1.45) | (0.22) |
| Ln Population | 0.72*** | 1.17 | 1.20 | 0.76*** |
|  | (0.05) | (0.24) | (0.36) | (0.05) |
| Republican Governor | 0.94 | 0.90 | 0.95 | 0.90 |
|  | (0.11) | (0.17) | (0.18) | (0.11) |
| Partisan Supreme Court Elections | 0.74* | 0.28* | 0.27* | 0.77 |
|  | (0.11) | (0.15) | (0.16) | (0.12) |
| Citizen Ideology/100 | 4.01 | 33.84 | 18.48 | 2.76 |
|  | (4.07) | (166.30) | (84.55) | (2.95) |
| South | 0.63** | 0.095 | 0.11 | 0.61** |
|  | (0.11) | (0.30) | (0.30) | (0.11) |
| Cumulative Executions*_t-1_*/10 |  | 0.00 |  |  |
|  |  | (0.01) |  |  |
| Executions*_t-1_*/10 |  |  | 0.00 | 0.03 |
|  |  |  | (0.00) | (0.17) |
| Constant | 96.54*** | 43.88 | 89.02 | 72.95*** |
|  | (0.92) | (3.27) | (3.58) | (0.93) |
| Year FE | Yes | Yes | Yes | Yes |
| *One or more Death Sentences* |  |  |  |  |
| Cumulative Death Sentences*_t-1_*/10 | 1.09*** |  |  | 1.08*** |
|  | (0.03) |  |  | (0.02) |
| Homicides*_t-1_*/100 | 1.00 | 0.98 | 0.98 | 1.00 |
|  | (0.02) | (0.02) | (0.02) | (0.02) |
| Racial Threat/100 | 3.02* | 1.36 | 1.19 | 2.91* |
|  | (1.44) | (1.20) | (1.16) | (1.40) |
| Lynchings/10 | 1.27*** | 1.31*** | 1.30*** | 1.27** |
|  | (0.09) | (0.08) | (0.08) | (0.09) |
| Ln Population | 1.91*** | 2.84*** | 2.86*** | 1.94*** |
|  | (0.08) | (0.15) | (0.17) | (0.09) |
| Republican Governor | 1.01 | 0.99 | 1.00 | 0.99 |
|  | (0.06) | (0.05) | (0.06) | (0.0) |
| Partisan Supreme Court Elections | 1.00 | 1.06 | 1.05 | 1.02 |
|  | (0.09) | (0.18) | (0.18) | (0.09) |
| Citizen Ideology/100 | 0.33 | 0.20 | 0.17 | 0.28 |
|  | (0.21) | (0.24) | (0.20) | (0.19) |
| South | 1.00 | 0.96 | 0.96 | 0.98 |
|  | (0.11) | (0.18) | (0.19) | (0.11) |
| Cumulative Executions*_t-1_*/10 |  | 1.06 |  |  |
|  |  | (0.04) |  |  |
| Executions*_t-1_*/10 |  |  | 2.06* | 0.82 |
|  |  |  | (0.67) | (0.42) |
| Constant | 0.00^***^ | 0.00*** | 0.00*** | 0.00*** |
|  | (0.00) | (0.00) | (0.00) | (0.00) |
| Year FE | Yes | Yes | Yes | Yes |
| Total obs. | 102,065 | 97,536 | 97,260 | 97,260 |
| Nonzero obs. | 5,057 | 4,835 | 4,835 | 4,835 |
| Log pseudolikelihood | -17,652.32 | -17100 | -1709.22 | -16774.99 |

***p < 0.001, **p < 0.01, *p < 0.05 Notes: Reported values are incidence rate ratios for the negative binomial model and odds ratios for the logit model. Robust standard errors clustered by county in parentheses. Each variable is rescaled by the factor indicated in order to generate coefficients that can be more easily interpreted. Models specified with year fixed effects.

## Supplemental File 2: Constructing the Homicide Database

The homicide database was constructed using two data sources—both the Centers for Disease Control (CDC) and the Federal Bureau of Investigation (FBI) provide counts of homicides over time for the entire nation. The CDC reports are based on death certificates filed with county health departments whereas the FBI numbers come from local police agencies. CDC numbers are consistently higher than FBI numbers because they list all victims of homicide, whether or not the police have solved the crime. Note that the CDC numbers are organized by the county of residence of the victim, whereas FBI numbers refer to the county of the crime. In spite of differences in reporting practices, the two series track closely with each other; populous counties have more homicides, no matter which indicator is used. A regression predicting the CDC number by the FBI number (with no constant term) shows that there are 1.0225 CDC homicides for every homicide recorded by FBI; the R^2^ for this one-variable equation is 0.9785.

The two series do not cover exactly the same time periods and have some gaps, so we take advantage of the high correlation between the two indicators to generate a single combined indicator based on the CDC numbers where available. Our final database is constructed as follows, with each estimate being used only if an estimate or observed value from the previous step was not available:

1. Direct observations based on CDC reports by county by year; approximately 37 percent.
2. Linear interpolation for individual missing observations where CDC observations are available for that county in contiguous years; approximately 1 percent.
3. Regression estimates of the CDC number based on FBI data; approximately 51 percent, particularly for counties with populations under 100,000 in the period after 1989, when the CDC stopped reporting such numbers.
4. Estimates of county values based on the typical share of total state homicides reported by the CDC, particularly for the period of 1989 through 2004. During this time the CDC reported state totals but not for individual counties, except for the largest counties. This affects approximately 4 percent of all observations.
5. Estimates of county values based on the typical share of total US homicides reported by the CDC, for the period of 2005 to 2019; this affects approximately 7 percent of all observations.
