# Supplementary material for: Learning to kill: Why a small handful of counties generates the bulk of US death sentences
Source: PLoS One. 2020 Oct 27;15(10):e0240401. doi: 10.1371/journal.pone.0240401 (PMC7591063; doi:10.1371/journal.pone.0240401)
Supplement: Attachment — Submitted filename: LearningToKill-RR-ResponseToReviewers.docx [file pone.0240401.s011.docx]

Bryan L. Sykes, Ph.D.
Academic Editor
PLOS ONE

PONE-D-20-20778
Learning to kill: Why a small handful of counties generates the bulk of US death sentences

August 28, 2020

Dear Dr. Sykes,

Thank you for the opportunity to revise our article, Learning to Kill. We were pleased that you and the reviewers found that the paper has merit, and we appreciate the questions and suggestions that you have made. Here, we enumerate the issues raised and provide our responses. We have also attached the requisite revised files, according to your correspondence of August 21.

We start with the comments you highlighted, then other comments by each reviewer.

*First, Reviewer 1 believes that it would be helpful to show the top 25 counties in terms of death sentences per homicide (along with the county homicide rate).*

- This was a very insightful comment; we have replaced Table 1 with a new version that also includes the death sentencing rate per 100 homicides, and the homicide rate per 100,000 population. These additions increase the power of the table, showing low correlations across the different variables. We’ve added text around the table to make this point clear. This was a very helpful suggestion and we think it enhances our points nicely. Thank you for that suggestion.

*Similarly, Reviewer 2 raises the question of how influential county death sentences are on the share of state executions.*

- We have tested a model now where we look at cumulative executions stemming from the county in question, as well as the lagged number of executions in the previous year, substituting (or adding) these for cumulative death sentences in our main model from Table 3. Results show that our original model is a better specification; we have added these alternative tables to the supplemental materials and written a footnote following table 3 to this effect.

*Second, Reviewer 1 questions whether this story is purely about event dependence or whether your findings are a result of "differences in preferences" for the death penalty.  Is there any way you can distinguish between these two conceptual frameworks (i.e., preference vs. dependence)?  One possible way to distinguish between these two competing frameworks would be if states or counties passed (or tried to pass) legislation on the death penalty, or if there are local policies governing the application of the death penalty.  If you find event dependence after controlling for such measures, this approach would strengthen your evidence about event dependency for Reviewer 1.*

- We incorporate “citizen ideology” into our model in Table 3. This variable varies over time as well as across the 50 states and distinguishes among conservative and progressive public opinion. Further, this variable is shaped empirically like a normal distribution. Indeed, some are more conservative than others, but there are no “far outliers” that would explain such a process. Indeed, Harris County, Texas, is less supportive of the death penalty in terms of public opinion survey responses than the rest of the state (see Baumgartner et al. 2018, p. 285). We’ve added a footnote to this effect at the bottom of p. 9 in the main text.
- With regards to possibly measuring local-level variability in laws, because criminal justice is a state function, there are no relevant county-level policies. (Local ordinances may affect some things, but not homicide.) At the same time, of course, the local District Attorney plays a key role. Our modelling strategy has been to assess these locally varying characteristics (e.g., homicides, history of lynchings, racial dynamics, population size) and then see if we see a self-reinforcing process, which we do. We have added a footnote before Table 3 to make these points more clear.

*Third, at the conclusion of your article, I was left with the "so what can be done" question.  If this is a story about event dependency, what must counties do to break such dependency, or are some counties a perpetual lost cause?  Simply put, what are your policy prescriptions?*

- We believe there are several reasonable policies, most importantly electing new DAs, and we have noted how often this has occurred, including in the highest-use counties, Philadelphia, PA and Harris, County TX, both of which elected DAs in the 2016 elections who have pledged not to seek the death penalty. Further, public opinion changes significantly over time, as is captured in our year FEs. In fact, there is a lot of variability over time. We have added relevant text in the conclusion, and we end the paper now with this very important caveat. Thank you for that reminder.

Reviewer 1 had some other points, which we have addressed as follows:

- *New York City death sentences:* Indeed several of the New York cases were from the city, and others from near suburbs. We’ve revised the text to delete reference to this question; there were 2 in Kings County, 1 in Queens, which are in the City, and 3 in Suffolk and 1 in Westchester, which are near suburban counties. Thanks to the reviewer for catching this error.
- *More intuition on the measure of Racial threat.* We’ve added relevant text in a footnote just after the first mention of this variable, before Table 2.
- *The Governor of the state may be less important than the Mayor of the large city*. While we can find examples either way, the death penalty is indeed a state function so we have not revised the text on this matter. We have, however, added a footnote before Table 3 to clarify.
- *Google counts of searches for the n-word*: We can’t do this for the full time period so have not taken this on.

In all, we thank you for the opportunity to make these improvements, and believe that these have significantly enhanced the quality of our paper. We will look forward to hearing from you.

xxx

Corresponding author
